# Supplementary material for: Time-course analysis of liver and serum galectin-3 in acute liver injury after alpha-galactosylceramide injection
Source: PLoS One. 2024 Feb 8;19(2):e0298284. doi: 10.1371/journal.pone.0298284 (PMC10852258; doi:10.1371/journal.pone.0298284)
Supplement: Attachment — Submitted filename: Response to Reviewers.docx [file pone.0298284.s002.docx]

**Response to the Reviewer**

------------------------------------------------

**To Reviewer #1:**

- *This paper follows galectin-3 expression, fibrosis and inflammatory markers in liver and serum after injection with aGalCer in mice. The focus on an early time course and a very specific type of liver injury is very interesting and worthwhile. The authors also compare with two chronic models: NASH and primary sclerosing cholangitis (PSC). The data are convincing and interesting, and well discussed. However a main point and some smaller need to be addressed.*

**Response:** We sincerely appreciate your thoughtful comments and extend our heartfelt gratitude for reviewing our manuscript.

- *The number of mice used needs to be mentioned for each experiment, e.g. in figure and table legends, and also what the error shown (+/- in Table, bars in figures) is, e.g. SD or SEM. and statistics.*

**Response:** We listed the number of the mice used in each experiment as "n" in Table (page 12 in the revised version). And we also add the number of mice for each group in figure legend. It was mentioned in the "Statistics" section that ± in the table is SD, but to make it clearer, we added it to the legend of the Table (page 12 line 247-248 in the revised version). The statistics used were also mentioned in the "Statistics" section, but to make it clear, were added to the legend of each figure. (page 14 line 290-293, page 14 line 310-314, page 14-15 line 332-336, in the revised version)

- *As this reviewer understands, aGalCer was injected ip only once, and then the mice followed. Does it give liver injury every time ? Or were there any cases of no (or too little effect) and were these also included in the results, or were they deleted as failed (no take) experiments?*

**Response:** α-GalCer-induced acute liver injury is a very stable model of liver injury, most of which is induced by a single intraperitoneal injection. In our study, all mice treated with α-GalCer were included in the data. We have added this to the site of the description of GalCer in the "Introduction" (page 5 line 105-106 in the revised version): and at the beginning of the "Results" section (page 10 line 220 in the revised version).

- *In Fig 3, 4A and B and 5 B and C it would be better to show mean and individual data pouts instead of error bars as now.*

**Response:** We have changed Fig 3, 4A and B and 5 B and C from mean values and error bars to mean values and individual data pouts.

- *The correlation in Fig. 4C is not very striking and convincing, but worth showing more for the lack of correlation.*

**Response:** Regarding the finding of only a weak correlation between serum Gal-3 and serum ALT levels, we hypothesized that while ALT is a hepatic lysosomal enzyme that is elevated by hepatocyte destruction, the timing of the response of serum levels to Gal-3, which is thought to be elevated by factors other than hepatocyte destruction, may be different. We have added the following sentence in “Discussion”: (page 18 line 391-397 in the revised version): “Only a weak correlation was found between serum Gal-3 level and serum ALT levels. This may be related to the fact that ALT is a hepatic enzyme that is elevated by the destruction of hepatocytes. Serum Gal-3 and serum ALT, both are marker which elevated in acute liver injury, serum ALT level has the property of being elevated when hepatocyte destruction occurs. In contrast, serum Gal-3 level may be derived from the fact that it is elevated not only by hepatocyte destruction but also by other factors that precede inflammation and destruction.”

- *In head of Table 1, it says ug/ml for serum Gal-3, but should perhaps be ng/ml as said in legend?*

**Response:** We sincerely apologize for the error in the text. "ug/mL" is correct. We have corrected the error in the legend. We sincerely appreciate your comment.

- *Some more recent up to date overview of galectins would be nice to include in reference list besides Ref 1-3.*

**Response:** We added four new references. We mentioned the physiological role of galectins, galectin typing and structure. We also mentioned that galectins have recently received attention not only in the field of inflammation and fibrosis, but also in the field of cancer research and therapy. We have added the following sentence in “Introduction”: (page 3 line 51-67 in the revised version): Galectins are widely expressed in various cell types. Their roles are diverse and include intercellular and cell-matrix adhesion, regulation of cell proliferation, apoptosis, mRNA splicing, signal transduction, and immune regulation [3]. Galectins' roles in angiogenesis, cell migration, and tumor immune evasion during carcinogenesis are also closely related to cancer biology, and galectins have recently attracted much attention in the field of cancer research and therapy [4, 5]. Galectins, soluble proteins, play important regulatory roles inside and outside the cell. Intracellular galectins exhibit functions such as regulating cell proliferation and apoptosis and regulate intracellular signaling pathways through protein-protein interactions [6]. Galectins secreted extracellularly bind to receptor proteins and act as mediators of inflammation, for example, or are involved in cell adhesion. Galectins are classified into three groups based on the composition of their intramolecular sugar-binding domains: proto-types, which have one sugar-binding domain and form dimers; chimeric types, which have a sugar-binding domain and a multimeric domain and form multimeric bodies; and tandem repeat types, which have two sugar-binding domains.

The four references we added were re-numbered and listed under "References" as 3-6 (page 20-21 line 432-444 in the revised version).

Again, thank you for giving us the opportunity to strengthen our manuscript with your valuable comments and queries. We have worked hard to incorporate your feedback and hope that these revisions will satisfy your concerns and permit you to recommend our submission for publication.
